# Repetitive syncope caused by a rare massive sporadic malignant peripheral nerve sheath tumor involving carotid arteries: A case report

**DOI:** 10.1097/MD.0000000000024386

**Published:** 2021-02-05

**Authors:** Tiehao Wang, Jiarong Wang, Jichun Zhao, Ding Yuan, Wenqing Yao

**Affiliations:** a Department of Vascular Surgery; b Department of Pathology, West China Hospital, Sichuan University, Chengdu, China.

**Keywords:** carotid artery, malignant peripheral nerve sheath tumors, neck mass, neuroendocrine activity, syncope

## Abstract

**Rationale:**

Malignant peripheral nerve sheath tumors (MPNSTs) are rare sarcomas arising from peripheral nerves. MPNSTs are uncommon in the head and neck, and various clinical manifestation often make the diagnosis challenging.

**Patient concerns:**

A 67-year-old female was referred for evaluation of repetitive syncope with a massive mass in the neck. Preoperative evaluation revealed potential neuroendocrine activity of the mass and enhanced computed tomography showed carotid artery was involved.

**Diagnosis:**

According to the preoperative imaging, intraoperative finding and postoperative pathological examination, the diagnosis of left neck MPNST involving left carotid arteries was made.

**Interventions:**

Volume expansion therapy with phenoxybenzamine started one week before surgery. Complete surgical resection of the mass was performed and pathological analysis suggested the diagnosis of MPNST. The postoperative radiotherapy was not given due to her poor nutrition.

**Outcomes:**

This patient recovered well after surgery and no sign of recurrence was noted at 2-year follow-up.

**Lessons:**

Though the involvement of carotid artery with neuroendocrine activity is rare in sporadic MPNST, preoperative scanning of blood and urine catecholamine is crucial for intraoperative hemodynamic stability, especially when carotid artery is involved.

## 1 Introduction

Malignant peripheral nerve sheath tumors (MPNSTs) are uncommon and biologically aggressive sarcomas which originate from peripheral nerves or from cells associated with the nerve sheath. As MPNSTs can arise from multiple cell types similar with other spindle cell sarcomas, the various clinical manifestation and histopathological appearance often make diagnosis and classification somewhat challenging.^[1,2]^ In addition, the prognoses are generally poor, attributed to high rates of recurrence in the early stage and low response rate to chemotherapy in advanced stages.^[3]^ MPNSTs accounts for approximately 2% of all sarcomas, with an estimated incidence of 5 people per million per year.^[4]^ Compared to other genomically complex sarcomas, MPNSTs tend to present earlier in life and are mostly prevalent between 20 and 50 years.^[5]^ Though large peripheral nerves in the extremities (i.e., sciatic nerve, brachial plexus, etc.) are typically affected, it was reported that, in rare conditions, 4% of the primary MPNSTs can arise in the head and neck.^[6]^ We report a case of huge primary malignant peripheral nerve sheath tumor in the neck with carotid artery involvement, presenting with repetitive syncope and negative local nerve symptoms.

## 2 Case report

A 67-year-old female was initially seen in local hospital for evaluation of repetitive dizziness 10 years ago, and a 1∗2 cm small mass was found in her left neck by ultrasound. After initial diagnose with hypertension, standard antihypertensive drugs were prescribed. Due to its silent character and small diameter, the mass was left for surveillance. The symptom was temporarily relieved and the patient did not come back during the follow-up until one year before admission to our hospital, the mass in the left neck started growing rapidly and repetitive syncope occurred.

**Figure 1:**
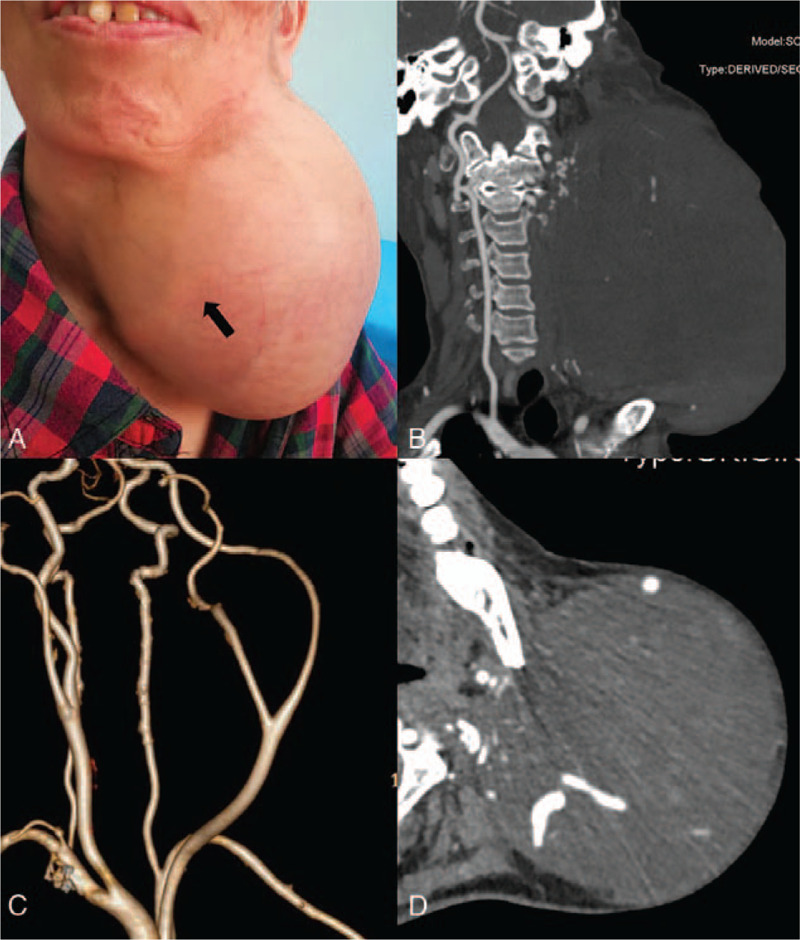
(A) Appearance of the front view of the mass in the neck, with “Y” shape carotid artery bifurcation visible (arrow). (B) Coronal view of the computed tomography angiography (CTA) of the patient. (C) CTA showed carotid arteries were compressed and translocated by the mass. (D) Axial view of CTA revealed the left carotid artery was involved by the mass.

On admission, the patient complained of left frontier pain and repetitive syncope, with negative local nerve dysfunctions (i.e., hoarseness, difficulty in swallowing). No signs or symptoms of neurofibromatosis type 1 (i.e., cafe au lait spots, Lisch nodules, neurofibromas, bone deformities, etc.) was noted. Physical examination revealed a huge mass in the left neck, with the “Y” shape carotid artery bifurcation visible (Fig. 1A). The computed tomography angiography (CTA) showed the diameter of the mass was 20cm∗17cm∗15 cm, with the upper border reaching the level of temporomandibular joint and the lower border reaching the upper chest wall (Fig. 1B); left common carotid artery (CCA), internal carotid artery (ICA) and external carotid artery (ECA) were compressed and translocated by the mass (Fig. 1C); no obvious boundary between arteries and the mass (Fig. 1D). Due to repetitive syncope and relatively poor physical condition, neoadjuvant radiation or chemotherapy was inapplicable and surgical removal of the mass was assessed. Preoperative blood test revealed an elevated level of urinary norepinephrine at 101.97 μg/24 h urine (16.3–41.5 μg/24 h urine), which suggested potential neuroendocrine activity. Thus, volume expansion therapy with phenoxybenzamine started one week before surgery.

**Figure 2:**
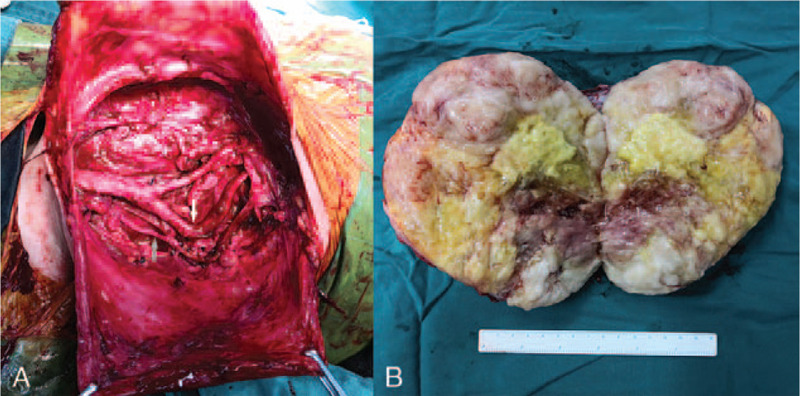
(A) Surgical view of the neck after the mass was completely resected. White arrow indicated left internal carotid artery; gray arrow indicated left vagus nerve. (B) Cross-section of the resected mass.

A longitudinal incision was adopted and the mass was gradually mobilized by blunt dissection. Change of blood pressure was noted when manipulating the mass. Left common carotid artery (CCA) and external carotid artery (ECA) were totally wrapped in the mass and internal carotid artery (ICA) was partially involved. After intravenous usage of heparin, intermittent clamp of the CCA facilitated dissection and mobilization of the CCA and carotid bifurcation following the proximal-distal route. Careful dissection and control of the ICA and ECA were obtained from the distal part. The involved vessel walls were partially excised and repaired by 6–0 prolene. The nourishing vessels from ECA were ligated and the mass was further mobilized and completely excised after careful dissection from the vagus trunk (Fig. 2A). The patient stayed in intensive care unit for 2 days and postoperative urine norepinephrine returned to normal level. The patient was discharged on postoperative day 5 without any adverse events.

**Figure 3:**
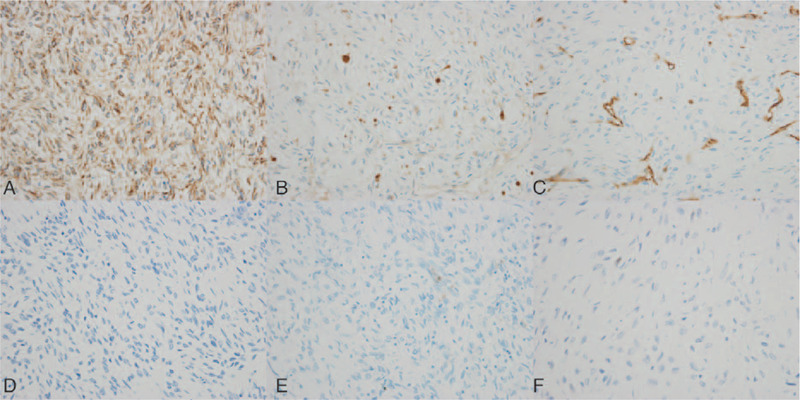
Immunohistochemical staining images of the mass. (A) strong positive staining of nestin (×400). (B) focal positive staining of S-100 (×400). (C) focal positive staining of CD34 (×400). (D) negative staining of SMA (×400). (E) negative staining of HMB-45 (×400). (F) negative staining of MYOD1 (×400).

**Figure 4:**
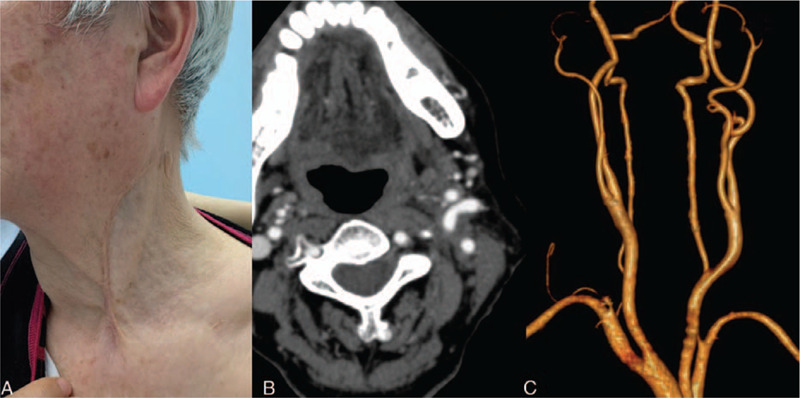
Two-year follow-up of this patient. (A) Appearance of the front view of the neck. (B) Axial view of computed tomography angiography (CTA) of previous operative region. (C) CTA showed carotid arteries were patent and distorted.

The cross section of the mass showed pleomorphic pattern (Fig. 2B) and pathological analysis of the specimen revealed spindle cell sarcoma with myxoid degeneration and necrosis, accompanied with focal observation of rhabdomyoblast-like cell and chondroid differentiation. Immunohistochemical staining demonstrated strong positivity of nestin and focal positivity of S-100, CD34 and myoD1, while STAT6, desmin, myogenin, SMA, CR, EMA, HMB45, β-C, ALK-1 immunostaining were all negative (Fig. 3). The histomorphology and results of immunohistochemical staining supported the diagnosis of MPNST. Given the potential benefit from adjuvant radiotherapy in MPNST, the patient was referred to cancer radiotherapy outpatient clinic, however, the patient did not receive the regimen due to poor nutrition. At 2-year follow-up, the patient had no signs of nerve symptom and tumor recurrence, and CTA showed carotid arteries were distorted and patent (Fig. 4). Moreover, syncope or dizziness didn’t occur during the follow-up.

## 3 Discussion

The causes of syncope are diverse, among which malignancy in head and neck is relatively rare, less than one in 250 patients in a screen of approximately 4500 cases.^[7]^ Malignancy-related syncope is commonly attributed to involvement of vasodepressor or vagal reflex arch, which were reported in pharyngeal or laryngeal carcinoma or carotid body tumor.^[7,8]^ By comparison, few evidence is available regarding the relationship between MPNST and syncope. MPNSTs are groups of soft tissue sarcomas that originate from peripheral nerves or differentiate along the lines from various elements of the nerve sheath (i.e. fibroblast, schwann cells, etc.), commonly presented in large peripheral nerves in the extremities and trunks. Unlike benign schwannomas, MPNSTs are seldom found in the head or neck area, comprising only 4% of all head and neck sarcomas.^[9]^ MPNSTs in the head and neck (HN-MPNSTs) can be either sporadic or associated with neurofibromatosis type 1 (NF1), an autosomal dominant disorder affecting neurofibromin 1 suppressor gene.^[3,10]^ It was reported that 20% MPNST patients were accompanied with neurofibromatosis and MPNST occurred in 40% to 50% of patients with neurofibromatosis,^[6]^ but our case did not find any signs or symptoms of neurofibromatosis. Published case series of HN-MPNSTs suggested the most common onset symptom was a painless, rapidly growing cervical mass without nerve palsy,^[11]^ while local compression or nerve symptoms may also occur, like airway obstruction, dysphagia and hoarseness.^[12]^ However, sporadic MPNST with a long silent period followed by a rapid enlarging period is less common, involvement of carotid bifurcation causing repetitive dizziness and syncope as the onset syndrome is exceptionally rare.

It is known that MPNSTs can present in various differentiation patterns, generally characterized by commutative hypo- and hyper-cell areas or asystematic growth pattern of spindle-shaped cells.^[13]^ Besides, epithelioid or other heterogeneous components could be found in approximately 15% of MPNSTs,^[14]^ other components consisted of rhabdomyoblasts,^[14,15]^ cartilaginous,^[16]^ osseous,^[17]^ smooth muscle,^[17]^ neuroendocrine and liposarcomatous components.^[16]^ A single MPNST rarely has two or more different differentiations,^[18]^ and our case presented two heterogeneous components, namely, rhabdomyoblasts and cartilaginous components. Interestingly, no glandular or neuroendocrine differentiation component was observed in pathological analysis, but preoperative urine catecholamine test showed a high level of norepinephrine, indicating potential neuroendocrine activity of the tumor. Thus, we scheduled volume expansion therapy assisted with phenoxybenzamine one week before the surgery, which reduce the risk of dramatic change of intraoperative blood pressure when manipulating the mass. It was reported that neuroendocrine differentiation was rare and only observed in glandular MPNSTs in vast majority of instance.^[15]^ Our case suggested preoperative test of blood and urine catecholamine is necessary to identify potential neuroendocrine activity of MPNST, in case of sudden change of blood pressure during the surgery, which is especially catastrophic when the tumor involved carotid artery bifurcation.

The diagnosis of MPNST simply based on histomorphology was difficult to differentiate from other sarcomas. Some neural markers, like S-100 and CD56, were proved to be sensitive markers for peripheral nerve sheath tumors.^[15]^ S-100 was classically regarded as the best marker for MPNST and was positive in about 50% to 90% of the tumors.^[19]^ Recent studies proposed combined immunostaining of nestin could reach a high sensitivity than other neural markers in MPNST.^[20]^ And focal S-100 positivity help to differentiate from schwannoma which show diffuse S-100 positivity.^[21]^ In addition, combination staining with SMA and HMB-45 can assist in distinguishing MPNST from leiomyosarcoma and malignant melanoma, respectively.^[19]^ In certain circumstances, MPNST with glandular differentiations or neuroendocrine activity should be distinguished from other soft tissue tumors with dual differentiation components like synovial sarcoma, and immunostaining nestin/S-100 yielded high specificity and positive predictive values.^[15]^

Current first-line treatment option for MPNST is surgery, regardless of primary or recurrent lesion. According to current consensus, the mass in our case would be considered as unresectable due to large diameter and carotid artery involvement. Usually in that case, radiotherapy or chemotherapy was considered as the only option. However, vascular surgical technique has enabled the surgeons to control and dissect vessels, and when necessary, partially resect involved vessels and then repair or reconstruct with autogenous veins, thus complete resection of the mass became possible. In our case, dissection and control of proximal CCA was firstly obtained, then intermittent clamp of CCA facilitated dissection and control of ICA. Groin and thigh were also prepped and draped in case harvest of saphenous vein is needed. As MPNSTs are rare and in lack of related evidence, their optimal perioperative management still remains controversial, mainly focusing on the role of neoadjuvant therapy and adjuvant therapy.^[22,23]^ The mainstream opinion suggested adjuvant radiotherapy in MPNSTs with large dimensions or aggressive histology should be considered.^[22]^ However, a retrospective analysis of national cancer registry compared 324 HN-MPNSTs and 1680 MPNSTs at other body sites (other-MPNSTs) from 1973 to 2012, the results showed combination of surgery and radiotherapy can contribute to better survival in other-MPNSTs but not in HN-MPNSTs.^[24]^ The study also revealed average tumor size for HN-MPNSTs was 4.9 cm, compared with 8.7 cm for other-MPNSTs, and HN-MPNSTs had a higher 5-year disease-specific survival than other-MPNSTs (65.1% vs 57.4%).^[24]^ Therefore, though MPNSTs in the head and neck area share the same histology, these certain groups of tumors may have specific clinical pattern and prognosis. Our case also did not undergo adjuvant radiotherapy and no recurrence was observed at 2-year follow-up.

Involvement of carotid artery with neuroendocrine activity is a rare manifestation of sporadic MPNST. Early diagnosis based on CT scan and biopsy is important to schedule perioperative management plan. Though a minority of HN-MPNSTs have neuroendocrine activity, preoperative scanning of blood and urine catecholamine is crucial for intraoperative hemodynamic stability. In addition, evidence concerning the role of adjuvant radiotherapy in HN-MPNSTs is inadequate.

## Author contributions

**Conceptualization:** Jichun Zhao, Ding Yuan.

**Data curation:** Tiehao Wang, Jiarong Wang.

**Investigation:** Tiehao Wang, Wenqing Yao.

**Supervision:** Ding Yuan.

**Writing – original draft:** Tiehao Wang, Jiarong Wang.

**Writing – review & editing:** Tiehao Wang, Jiarong Wang, Jichun Zhao.
